# Insulin-Like Growth Factor-1 Signaling Regulates miRNA Expression in MCF-7 Breast Cancer Cell Line

**DOI:** 10.1371/journal.pone.0049067

**Published:** 2012-11-30

**Authors:** Elizabeth C. Martin, Melyssa R. Bratton, Yun Zhu, Lyndsay V. Rhodes, Syreeta L. Tilghman, Bridgette M. Collins-Burow, Matthew E. Burow

**Affiliations:** 1 Department of Medicine-Section of Hematology and Medical Oncology, Tulane University, New Orleans, Louisiana, United States of America; 2 Department of Pharmacology, Tulane University, New Orleans, Louisiana, United States of America; 3 College of Pharmacy, Xavier University of Louisiana, New Orleans, Louisiana, United States of America; Baylor College of Medicine, United States of America

## Abstract

In breast carcinomas, increased levels of insulin-like growth factor 1 (IGF-1) can act as a mitogen to augment tumorigenesis through the regulation of MAPK and AKT signaling pathways. Signaling through these two pathways allows IGF-1 to employ mechanisms that favor proliferation and cellular survival. Here we demonstrate a subset of previously described tumor suppressor and oncogenic microRNAs (miRNAs) that are under the direct regulation of IGF-1 signaling. Additionally, we show that the selective inhibition of either the MAPK or AKT pathways prior to IGF-1 stimulation prevents the expression of previously described tumor suppressor miRNAs that are family and cluster specific. Here we have defined, for the first time, specific miRNAs under the direct regulation of IGF-1 signaling in the estrogen receptor positive MCF-7 breast cancer cell line and demonstrate kinase signaling as a modulator of expression for a small subset of microRNAs. Taken together, these data give new insights into mechanisms governing IGF-1 signaling in breast cancer.

## Introduction

One of the hallmarks of breast cancer progression is the transformation of cancer cells to a more aggressive and metastatic phenotype through the initiation of hormone-independent growth and cell survival. Increased activity of both MAPK and PI3K/AKT signaling pathways is known to contribute to the progression and metastatic capabilities of breast tumors [1]. Both signaling pathways are activated through ligand binding of receptors that are responsive to growth hormones such as the tyrosine kinase insulin like growth factor receptor (IGF1R) [2]. Once bound to the IGFR1, insulin-like growth factor-1 (IGF-1) acts as a mitogen, augmenting tumor progression through the dual activation of these pathways. Through the transduction of its signal, IGF-1 acts as both a survival factor protecting cells from apoptosis and as a growth factor initiating differentiation and proliferation [3], [4]. Signaling through these pathways results in an increase in transcription of cell cycle genes, resulting in increased cellular proliferation. At the same time, the IGF signal induces an escape from programmed cell death through a combination of inhibition of pro-apoptotic genes and an increase in cell survival genes [5].

microRNAs (miRNAs) are small non-coding RNAs that regulate gene expression through degradation of mRNA or translational inhibition of target mRNA [6]. miRNAs act as key mediators in the progression and transformation of neoplasms through their regulation of proliferation, differentiation, and apoptosis [7], [8]. While many aspects of miRNA induced protein regulation are known, there is a growing need to uncover the complex and not completely understood regulatory mechanisms governing the activation and suppression of miRNA expression. The expression of many miRNAs is regulated by signaling molecules, including growth factors and endocrine molecules [9]–[12]. IGF-1 increases tumorigenesis via the activation of signaling cascades that favor cell proliferation, survival, and invasion [4], [5]. The tumorigenic effects employed by this signaling molecule may be attributed to the regulation of miRNAs harboring oncogenic or tumor suppressive capabilities. Recently, IGF-1 induction of AKT isoforms was demonstrated to alter miRNA expression patterns in murine lung fibroblasts [13]. The effects of IGF-1 induced signaling cascades on miRNA expression in estrogen receptor (ER) positive (+) breast carcinomas has yet to be evaluated. Uncovering mechanisms that govern miRNA expression defines a new layer in breast cancer biology that may give greater insight into pathways facilitating tumorigenesis.

In this study we set out to define miRNAs that are regulated by IGF-1 through signaling in the ER^+^ MCF-7 breast cancer cell line. Here we reveal through microarray several previously well defined oncogenic and tumor suppressor miRNAs which undergo significant changes in expression following IGF-1 treatment. Additionally, we show through the use of MEK and PI3K specific inhibitors, that tumor suppressive miR-15 and let-7 family members miR-195, let-7c, let-7g, and miR-98 are under the regulation of both MAPK and PI3K/AKT signaling, while miR-15b is solely regulated by MAPK signaling. These results define a role for kinase regulated miRNA expression as potential mediators of IGF-1 signaling in the ER^+^ breast cancer cells.

## Results

### IGF-1 regulates miRNA expression in MCF-7 breast cancer cells

**Figure 1:**
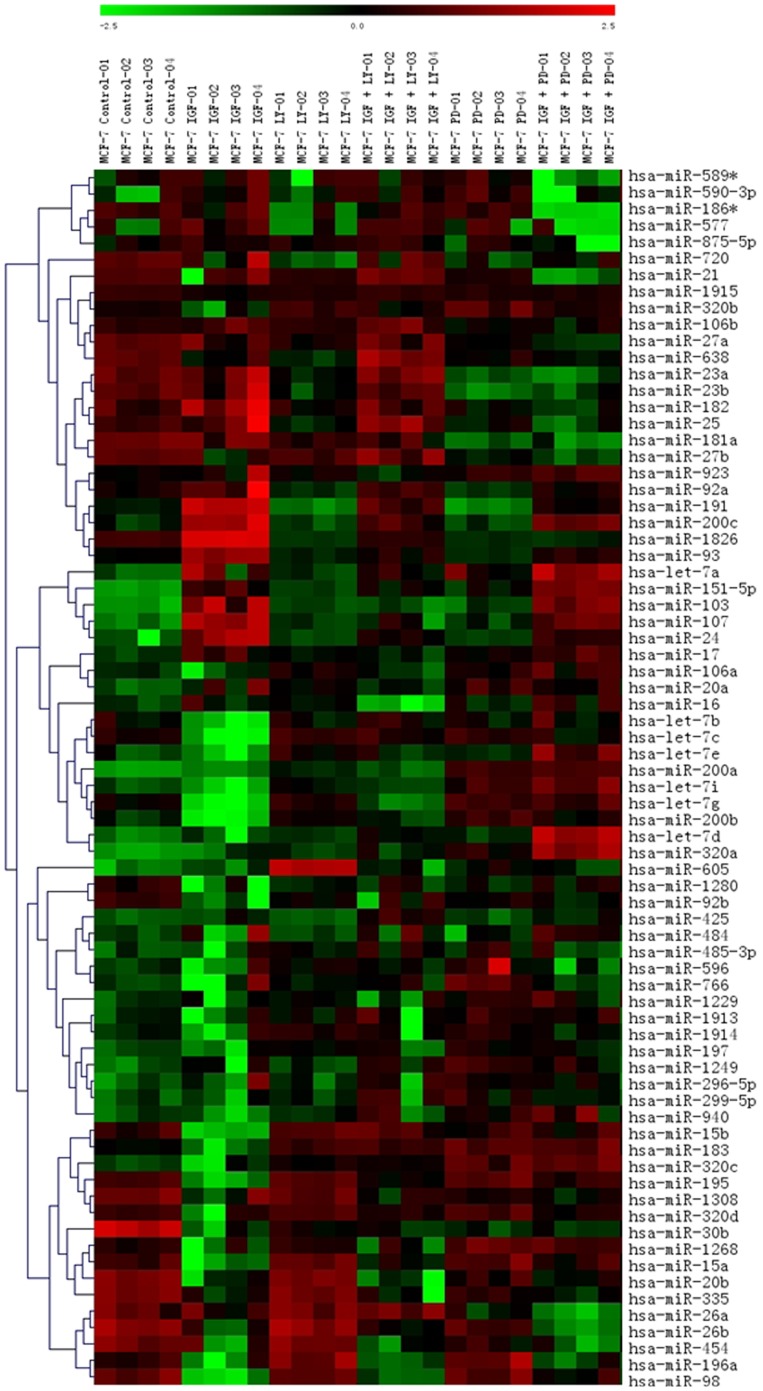
IGF-1 induced miRNA expression in MCF-7. Microarray analysis was performed on MCF-7 cells. Cells were grown in 5% charcoal stripped DMEM for 48 hours. Cells were then treated with 50 ng/mL IGF-1, 5 µM LY294002 or 10 µM PD98059 for 18 hours. Cells with combined IGF-1/LY294002 or IGF-1/PD98059 were pre-treated with inhibitor for 30 minutes followed by 18 hours of stimulation with IGF-1. Results represent quadruplicate internal repeats.

**Figure 2:**
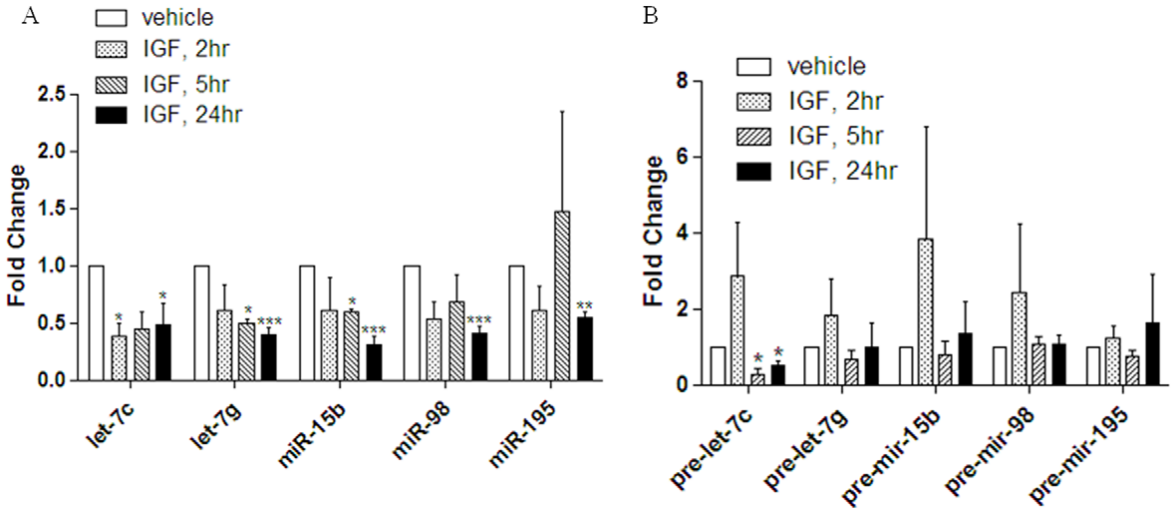
IGF-1 Represses miRNA Expression. MCF-7 cells were grown in 5% charcoal stripped DMEM for 48 hours. Cells were treated for 2, 5, or 24 hours with 50 ng/ml IGF-1 or vehicle. Cells were collected for total RNA extraction and q PCR was performed for (A) total miR-let-7c, let-7g, miR-15b, miR-98, and miR-195 and (B) pre-let-7c, pre-let-7g, pre-mir-15b, pre-mir-98, pre-mir-195. Normalization was to U6 and vehicle treated cells designated as 1. Error bars represent SEM, n≤5. * Significantly different from IGF, p≤0.05.

The effects of IGF-1 on oncogenic and tumor suppressive miRNAs in ER^+^ breast carcinomas have yet to be elucidated. Therefore, we performed a miRNA microarray to determine the effects of IGF-1 treatment on miRNA expression in the ER^+^ MCF-7 breast carcinoma cell line. A small subset of miRNAs undergo altered expression following 18 hours of treatment with 50 ng/ml IGF-1 compared to vehicle treated cells (Figure 1). Notably some previously defined oncogenic miRNAs (103/107, 1826, 191, 93) show significant increases in expression while known tumor suppressive miRNAs (15b, 98, 195, 200b, let-7c and let-7g) are significantly repressed with IGF-1 treatment (Table 1) [14]–[22]. This indicates that IGF-1 may stimulate tumorigenesis through regulation of miRNAs. Interestingly, the array data demonstrate that IGF-1 negatively alters levels of the tumor suppressor let-7 (let-7c, let-7g, and miR-98) and miR-16 (miR-195 and miR-15b) family members. To confirm microarray data we used qPCR to analyze miRNAs miR-15b, miR-195, miR-98, let-7c, and let-7g levels in MCF-7 cells following treatment with 50 ng/ml IGF-1 for 2 hours, 5 hours, and 24 hours. qPCR results revealed significantly decreased expression levels of all five tumor suppressor miRNAs (miR-195, miR-15b, let-7c, let-7g, and miR-98) following treatment with IGF versus that of vehicle treated cells. Let-7c was the only miRNA which demonstrated significant repression at 2 hours following IGF-1 treatment versus vehicle control (Figure 2). To gain better insight into possible mechanisms of IGF-1 regulation of these specific miRNAs, qPCR was performed for pre-let-7c, pre-let-7g, pre-mir-15b, pre-mir-98, and pre-miR-195 following 2, 5, and 24 hours of treatment with IGF-1. Following qPCR pre-let-7c demonstrated a significant decrease in expression following treatment with IGF-1 versus vehicle at both 5 and 24 hours (Figure 2). Pre-let-7g, pre-mir-15b, pre-mir-98, and pre-mir-195 showed no significant change in levels of expression following treatment with IGF versus vehicle at any time point (Figure 2). These data suggest that IGF-1 may be acting both transcriptionally and affecting miRNA biogenesis.

**Table 1:** miRNA Expression Altered by IGF-1 Treatment in MCF-7 Breast Cancer Cells.

miRNA	IGF	LY294002	PD98059	IGF/LY	IGF/PD
Let-7c	−1.75	-	-	-	-
Let-7g	−1.91	-	1.13	-1.36	-
miR-15b	−2.08	-	-	-	-
miR-195	−6.59	-	-	−1.71	-
miR-200b	−1.77	-	1.31	-	-
miR-30b	−5.39	−3.82	−4.53	−3.92	−4.38
miR-7	−6.87	-	−1.90	−3.84	−6.92
miR-98	−3.18	-	-	−2.14	-
miR-103	1.80	1.19	-	-	1.74
miR-107	1.75	1.15	-	-	1.54
miR-151-5p	1.79	1.30	1.48	1.56	2.19
miR-1826	2.83	−2.14	−2.03	−1.16	−1.96
miR-191	2.06	−1.39	−1.46	-	-
miR-200c	1.39	1.45	-	-	1.24
miR-93	2.25	−1.19	−1.27	-	-

Results represent fold change. “-” represents no significant change between vehicle treated MCF-7 cells and drug treatment.

### IGF-1 alters miRNA expression through the MAPK signaling pathway

**Figure 3:**
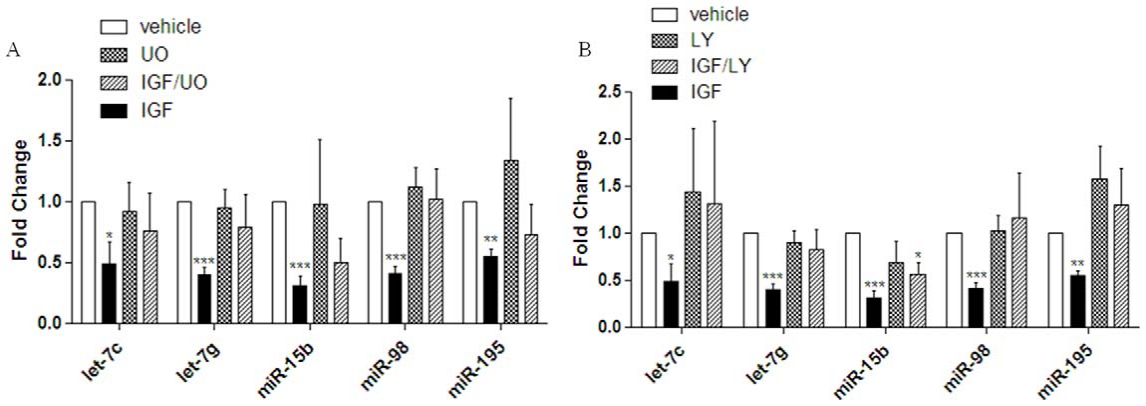
IGF-1 regulates miRNAs through MAPK and PI3K/AKT signaling pathways. (A) MCF-7 cells were grown in 5% charcoal stripped DMEM for 48 hours. Cells were treated with UO1261 for 30 minutes prior to 24 hours of treatment withvehicle. Q RT- PCR was performed for miRNAs miR-15b, miR-98, let-7c, let-7g, and miR-195. Normalization was to U6 and vehicle treated cells designated as 1. (B) MCF-7 cells were grown in 5% charcoal stripped DMEM for 48 hours. Cells were treated with LY294002 for 30 minutes prior to IGF treatment for 24 hours or vehicle. Normalization was to vehicle treated cells and error bars represent SEM, n≤5. * Significantly different from vehicle treated cells p≤0.05.

To delineate the signaling pathways involved in IGF-1 regulation of miRNAs, a microarray assay was performed in MCF-7 cells treated with 10 µM of the MEK1/2 inhibitor PD98059 for thirty minutes prior to treatment with IGF-1. The joint treatment of MCF-7 cells with IGF-1 (50 ng/ml) and PD98059 resulted in expression levels of some miRNA that were no longer statistically significant than that of vehicle, suggesting that some of the miRNAs altered by IGF-1 are under the regulation of MAPK signaling (Figure 1). To validate array data MCF-7 cells were treated for 24 hours with 50 ng/ml IGF-1, IGF-1+10 µM UO1261 (Mek1/2 inhibitor), or vehicle. Samples were analyzed by qPCR for alterations in mature miRNA sequences for miRNAs let-7c, let-7g, miR-15b, miR-98, and miR-195. Co-treatment of MCF-7 cells with IGF-1 and UO1261 resulted in miRNA expression levels that were no longer different than that of vehicle treated cells for all five tumor suppressor miRNAs (Figure 3A). Additionally, the sole treatment of MCF-7 cells with UO1261 had no significant effect on miRNA expression levels for miRNAs let-7c, let-7g, miR-15b, miR-98, or miR-195 compared to vehicle control (Figure 3A). These data suggest that IGF-1 induction of MAPK signaling regulates the let-7 family members (let-7c, let-7g, and miR-98) and miR-16 family members (miR-15b and miR-195).

### IGF-1 alters miRNA expression through the PI3K/AKT signaling pathway

To determine if IGF-1 regulation of miRNAs utilizes the PI3K/AKT signaling pathway, a microarray was performed using the PI3K inhibitor LY294002 prior to treatment with IGF-1 in the MCF-7 cell line. For miRNAs significantly altered by IGF-1 treatment, microarray data reveals that the joint treatment of MCF-7 cells with IGF-1 and 5 µM LY294002 resulted in expression levels equivalent to that of vehicle treated cells, indicating that IGF-1 may alter miRNA expression through the regulation of PI3K/AKT signaling (Figure 1). To confirm microarray data of IGF-1 PI3K/AKT regulated miRNAs the miR-16 and let-7 family members, miR-15b, miR-195, miR-98, let-7c, and let-7g, were chosen for further analysis. MCF-7 cells were treated for 24 hours with 50 ng/ml IGF-1, IGF-1+LY294002, or vehicle and were analyzed by qPCR for mature miRNA expression. While treatment of MCF-7 cells with IGF alone repressed let-7c, let-7g, miR-15b, miR-98, and miR-195 (Figure 2A), the co-treatment of MCF-7 cells with IGF-1 and LY294002 resulted the loss of miRNA repression and miRNA expression levels for miRNAs let-7c, let-7g, miR-98, miR-195 were no longer significantly different than that of vehicle. miR-15b was the only miRNA tested which was significantly repressed following co-treatment of IGF and LY294002 (Figure 3B). These data suggest that let-7c, let-7g, miR-98, and miR-195 are regulated by IGF through the PI3K/AKT pathway.

## Discussion

IGF-1 signaling is a mediator of both PI3K/AKT and MAPK signaling and has been demonstrated to enhance breast cancer tumorigenessis. While only a small subset of miRNAs is shown to be significantly regulated by treatment with IGF-1, some of these miRNAs appear to be family and cluster specific. The regulation of let-7 family members by PI3K/AKT and MAPK signaling is consistent with previous findings by Dangi-Garimella *et al* and Bhat-Nakshatri *et al* [9], [23]. Additionally both the miR-16 and let-7 families have previously been classified as tumor suppressors and target genes that are associated with EMT, apoptosis, and cell cycle progression (Table 2) [16], [17], [19], [24]–[26].

**Table 2:** miRNA families regulated by MAKP or PI3K/AKT signaling.

miRNA Family	miRNAs Altered	Pathway	IGF Regulation	Targets	Cellular Function
Let-7	miR-98, let-7c/g	AKT/MAPK	Down	HMGA2, Ras, Myc	Tumor Suppressor: Transformation, Proliferation
miR-16	miR-195, miR-195/miR-15b	AKT, MAPK	Down	Cyclin D1, CDK6, E2F3, VEGF, BCL2	Tumor Suppressor: Cell Cycle, Anti-angiogenic

The emerging importance of miRNAs in the regulation of proteins associated with proliferation, survival, and epithelial-to-mesenchymal transition (EMT) underscores the importance of uncovering mechanisms governing the expression of these small RNAs as a means to better understand the complicated processes of tumorigenesis and cancer cell progression [23], [24]. Determining the roles of endocrine molecules, growth factors, and signaling pathways in the regulation of miRNA expression is essential. Here we demonstrate for the first time a role for IGF-1 signaling in the regulation of miRNAs in the ER^+^ MCF-7 breast cancer cell line.

## Materials and Methods

### Cells and Reagents

MCF-7 human breast cancer cell line was acquired from American Type Culture Collection (Manassas, VA) and was maintained in Dulbecco's modified Eagle's medium (DMEM; pH 7.4; Invitrogen Corp., Carlsbad, CA) supplemented with 10% fetal bovine serum (Hyclone, Salt Lake City, UT), 1% Non-essential amino acids, minimal essential amino acids, sodium pyruvate, anti/anti, and insulin under mycoplasma-free conditions at 37°C in humidified 5% CO_2_ and 95% air as previously described [27].

### RNA Extraction and Quantitative Real Time RT-PCR

MCF-7 cells were grown in 5% charcoal stripped DMEM media and treated with 50 ng/ml IGF, 5 µM LY294002, 10 µM UO1261, or DMSO for 18 hours. Cells were harvested and total RNA extraction was performed using Qiagen miRNeasy RNA purification system according to the manufacturer's protocol. The quantity and quality of the total RNA was determined by measuring the absorbance at 260 and 280 nm using the NanoDrop ND-1000. 2 ug of total RNA was reverse-transcribed using the SA Bioscience RT 2 miRNA kit and qPCR was performed using U6, miR-15b, miR-98, miR-195, let-7g, and let-7c primers purchased from SA Biosciences and amplified n>3. Pre-miR PCR was performed using the miScript II Kit and miScript II SYBR green (Qiagen). Primers for pre-let-7c, pre-let-7g, pre-mir-15b, pre-mir-98, and pre-mir-195 were purchased from Qiagen. Data was analyzed using a student t-test and by comparing relative miRNAgene expression to U6 RNA. Relative gene expression was analyzed using 2-ΔΔCt method.

### MicroRNA MicroArray

MCF-7 cells were plated at a density of 2 million cells in 25 cm^2^ flasks in normal culture media (DMEM media supplemented with 10% FBS, 1% anti-anti, 1% essential amino acids, 1% non-essential amino acids and 1% sodium pyruvate) and allowed to adhere overnight at 37°C, 5% CO_2_ and air. The following day the media was changed to phenol red-free media (supplemented as above) and 5% charcoal stripped serum was substituted for the 10% FBS. Cells were treated with 50 ng/ml IGF, 5 µM LY294002, 10 µM PD98059, or vehicle control (DMSO) for 18 hours. Experiment was repeated for n = 3 biological repeats. Cells were harvested in PBS, collected by centrifugation, and total RNA extracted using the miRNeasy kit (Qiagen) according to manufacturer's protocol. Enrichment for miRNA was not performed. Quantity and quality of RNA was determined by absorbance (260, 280 nm). LC Sciences (Houston, TX, USA) performed Microarray assay using 5 µg total RNA which was size fractioned using a YM-100 Micron centrifugal filter (Millipore) and the small RNAs (<300 nt) isolated were 3′-extended with a poly(A) tail using poly(A) polymerase. Three biological samples of RNA were pooled and array was performed using quadruplicate internal repeats of pooled RNA and as previously described [28]–[30]. Data was analyzed by first subtracting the background and normalization of array was to statistical mean of all detectable transcripts. System related variation of data was corrected using LOWESS filter (Locally-weighted Regression) method [30]. Probes were single channel and detected signals greater than background plus 3 times the standard deviation was derived. P-values of the t-test were calculated were differentially detected signals were those with less than 0.01 p-values. Full array data including complete list of miRNAs measured and controls is available as supplemental material (Data S1). Data was analyzed using student t-test and ANOVA.

## Supporting Information

Data S1 **Full micro array data with a complete list of all miRNAs measured and controls used.**
(XLS)
